# Effects of high CD93 expression on tumor growth and angiogenesis in gastric adenocarcinoma

**DOI:** 10.1371/journal.pone.0355284

**Published:** 2026-08-12

**Authors:** Yujiao Dong, Panru Luo, Lili Chai, Xin Li, Jiawei Wang, Lingyu Wei, Jinsheng Wang

**Affiliations:** 1 Shanxi Provincial Center for Upper Gastrointestinal Cancer Research and Clinical Translation, Heping Hospital Affiliated to Changzhi Medical College, Changzhi, PR China; 2 Department of Pathology, Heping Hospital Affiliated to Changzhi Medical College, Changzhi, Shanxi, China; 3 Department of Pathology, Xi’an Central Hospital, Xi’an, Shaanxi, China; 4 Central Laboratory of Clinical Research, Heping Hospital Affiliated to Changzhi Medical College, Changzhi, Shanxi, China; 5 Department of Pathology, The First Clinical College of Changzhi Medical College, Changzhi, Shanxi, China; Longgang Otorhinolaryngology Hospital & Shenzhen Key Laboratory of Otorhinolaryngology, Shenzhen Institute of Otorhinolaryngology, CHINA

## Abstract

**Background:**

Gastric cancer remains a major cause of cancer-related mortality worldwide, with tumor recurrence and distant metastasis being primary contributors to poor prognosis; however, the underlying molecular mechanisms are not fully understood. CD93, a type I transmembrane glycoprotein, has been implicated in tumor angiogenesis and metastasis in various solid cancers, yet its role in gastric adenocarcinoma (STAD) requires further elucidation.

**Methods:**

We analyzed CD93 expression and its prognostic significance in STAD using TCGA and an independent cohort. CD93 was overexpressed or knocked out in SGC-7901 cells, with modulation efficiency confirmed by qRT-PCR and Western blot. Functional assays included CCK-8, colony formation, tube formation, endothelial permeability, and transendothelial invasion. A xenograft model using Ctr and sg-CD93 cells was established to assess tumor growth, with IHC performed for Ki67, CD34, and α-SMA.

**Results:**

CD93 was significantly upregulated in STAD tissues, and high expression correlated with poor patient survival. CD93 overexpression promoted cancer cell proliferation and enhanced tube formation in HUVECs. Interestingly, it also reduced endothelial monolayer permeability and inhibited transendothelial invasion of gastric cancer cells.

**Conclusion:**

CD93 facilitates gastric adenocarcinoma progression by promoting tumor cell proliferation and angiogenesis. Its dual role in enhancing tube formation while reducing endothelial permeability suggests a complex mechanism in tumor microenvironment regulation, highlighting its potential as a therapeutic target.

## Introduction

According to global cancer statistics, in 2022, the incidence and mortality rates of gastric cancer both ranked fifth among all cancers worldwide [1]. This has become a major issue that urgently needs to be addressed in the field of global public health [2]. Epidemiological data show that although the effective control of Helicobacter Pylori (HP) infection has reduced the global disease burden of gastric cancer, in developing countries, especially in China, the incidence rate of gastric cancer remains high and requires continuous attention and emphasis [3,4]. The vast majority of gastric cancer cases are of the adenocarcinoma type. Currently, the first-line treatment for gastric cancer still mainly consists of surgery and chemotherapy led by 5-fluorouracil. However, a considerable proportion of patients still have poor treatment tolerance and unsatisfactory therapeutic effects, which directly affects the clinical prognosis [5,6]. Therefore, clarifying the occurrence and development mechanism of gastric cancer and exploring new biomarkers or molecular targets have become key issues in the field of gastric cancer research [7].

Current research indicates that the interactions between tumor cells and various types of cells in the tumor microenvironment, particularly between parenchymal and non-parenchymal cells, play a significant role in tumor initiation and progression [8,9]. Among these, the vascular system, as a core component of the tumor microenvironment, is crucial for tumor advancement [10,11]. On the one hand, tumor cells exhibit the Warburg effect by mainly relying on glycolysis for energy supply, which leads to local tissue hypoxia and prompts tumor cells to secrete various pro-angiogenic factors and metabolic products, thereby promoting angiogenesis and meeting the nutritional requirements for tumor growth [12]; on the other hand, the interaction between tumor cells and vascular endothelial cells enables them to invade distant normal tissues through the vascular system [10,13]. Studies have shown that certain biomarkers related to angiogenesis and vascular permeability can be used for the diagnosis and treatment of metastatic cancer [14–16]. The mechanism of angiogenesis and vascular permeability in the occurrence and development of gastric adenocarcinoma still needs further exploration.

CD93, as a member of the C-type lectin XIV family, is widely expressed in various cell types including hematopoietic stem cells, trophoblast cells, endothelial cells, monocytes, neutrophils, B cells, platelets and natural killer cells [17]. CD93 is aberrantly overexpressed in a variety of malignancies and is frequently associated with adverse prognosis. In lung cancer, upregulation of CD93 correlates with lymph node and distant metastasis, and can impede antitumor immune responses by suppressing the migration of dendritic cells [18,19]. In gastric cancer, high expression of CD93 is closely associated with immune cell infiltration [20,21]. In colorectal cancer, hepatocellular carcinoma, and nasopharyngeal carcinoma, high expression of CD93 is also closely associated with increased risk of recurrence, immune infiltration, angiogenesis, and other malignant characteristics [22–24]. In leukemia, CD93 is specifically and highly expressed on leukemic stem cells and promotes their proliferation; blocking this pathway effectively suppresses leukemia progression [25–28]. In glioblastoma, CD93 affects patient survival by promoting angiogenesis and regulating the immunosuppressive microenvironment [29,30]. In breast cancer and osteosarcoma, CD93 promotes tumor proliferation, migration, and angiogenesis by activating the PI3K/AKT signaling pathway [31,32]. The above studies indicate that CD93 possesses multiple functions, including promoting angiogenesis, mediating immune evasion, and driving malignant proliferation, making it a potential tumor prognostic marker and molecular target. CD93 has now been demonstrated to be one of the key angiogenesis-associated genes in malignant tumors [33]. With the deepening of research, the function of CD93 as a key regulatory factor in the potential mechanism of malignant tumors has gradually been clarified. Most studies have pointed out that the CD93 protein plays an important role in cell adhesion, migration and angiogenesis, and the elevated expression of CD93 is positively correlated with poor prognosis in patients [24,34,35]. Consequently, CD93 holds promise as a novel target for anti-angiogenic therapy in solid tumors [34,36].

However, the specific mechanisms by which CD93 promotes angiogenesis and vascular permeability during the onset and progression of gastric adenocarcinoma remain unclear. To this end, we investigated the biological functions of CD93 in gastric adenocarcinoma cells and its effects on angiogenesis and permeability, aiming to identify new potential targets for the clinical treatment of gastric adenocarcinoma patients and provide innovative ideas for optimizing clinical treatment strategies.

## Methods and materials

### Data source and version

RNA-seq expression profiles of gastric adenocarcinoma and corresponding clinical survival data from the TCGA database (https://portal.gdc.cancer.gov/). Series matrix files from GEO datasets (GSE54129, GSE63089, GSE65801, GSE66229, GSE26901, GSE84433) were directly downloaded from the NCBI GEO database.

### Inclusion and exclusion criteria for cases

TCGA-STAD and GEO datasets: Primary gastric adenocarcinoma cases with complete survival information and CD93 expression data were included. Cases without survival time, unknown survival status, or follow-up duration of 0 were excluded; among these, cases with complete survival data were used for prognostic analysis.

### Cell lines and reagents

Human cell lines (gastric carcinoma cells SGC7901) was obtained from the Kunming Cell Bank of Type Culture Collection Chinese Academy of Sciences (Kunming, China). The cells cultured in high-glucose DMEM culture medium (Gibco, C11885500BT) supplemented with 10% fetal bovine serum (FBS) (VivaCell, C04001-500), penicillin (100 mg/mL), and streptomycin (100 mg/mL) and kept at 37℃ in a humidified atmosphere containing 5% CO_2_. The cell line was tested for mycoplasma contamination.

### Plasmids and sgRNAs

The full-length human CD93 cDNA (NM_012072) was cloned into the pcDNA3.1-EGFP-C2 vector to generate the CD93 overexpression plasmid (OE-CD93). The empty pcDNA3.1-EGFP-C2 vector was used as a negative control (OE#Mock). Both plasmids were synthesized by Hunan Tanyuan Biotechnology Co., Ltd. (Changsha, China).

For CRISPR/Cas9-mediated knockout, two high-scoring single-guide RNA (sgRNA) sequences targeting the CD93 gene were designed using the CRISPR design tool (http://crispr.mit.edu) and cloned into GFP-tagged CRISPR/Cas9 plasmid vectors. The sgRNA target sequences were: sgRNA-1, 5′-GATCGCCTTACTCTAACTGGCACA-3′; and sgRNA-2, 5′-GAGGCTGTCAGCTGAGGAGAGG-3′. A non-targeting sgRNA was used as the negative control (sg#Mock).

### Establishment of CD93-overexpressing cells

SGC-7901 cells were seeded in 6-well plates and cultured until reaching 60–80% confluence. Prior to transfection, the culture medium was replaced with serum-free and antibiotic-free RPMI-1640. For each well, 2.5 µg of plasmid DNA and 5 µL of P3000 reagent were diluted in 125 µL Opti-MEM. In a separate tube, 3.75 µL of Lipofectamine 3000 was diluted in 125 µL Opti-MEM. After 15 minutes of incubation at room temperature, the two mixtures were combined and added dropwise to the cells. After 8 hours, the transfection medium was replaced with complete culture medium. The resulting cell lines were designated as OE-CD93 (CD93 overexpression), OE#Mock (empty vector control), and NC (blank control).

### Establishment of CD93-knockout cells

SGC-7901 cells were seeded in 12-well plates and allowed to adhere for 4–6 hours until reaching approximately 70% confluence. For transfection, 1 µg of CD93-targeting CRISPR/Cas9-sgRNA plasmid together with the GFP reporter vector were diluted in 200 µL Opti-MEM, followed by the addition of 4 µL of linear PEI transfection reagent. The mixture was gently pipetted and incubated at room temperature for 10–15 minutes before dropwise addition to the cells. At 24 hours post-transfection, the medium was replaced with complete medium containing 2 µg/mL puromycin for selection. Selection was maintained for 24–48 hours until all surviving cells exhibited green fluorescence, confirming successful integration. Stable monoclonal knockout cell lines were expanded and designated as sg-CD93#1, sg-CD93#2, sg#Mock (non-targeting control), and NC (blank control).

### Validation of CD93 expression

The efficiency of CD93 overexpression and knockout was verified by quantitative real-time PCR (qRT-PCR) and Western blot. For qRT-PCR, total RNA was extracted using TRIzol reagent and reverse-transcribed into cDNA. qRT-PCR was performed using the following specific primers: CD93 forward, 5′-CCGGAAGTAACATTGAGGGCT-3′ and reverse, 5′-TCTGAGTCTCGTCCTTGTCAC-3′; GAPDH forward, 5′-GTGGTGAATGACACAGTTGG-3′ and reverse, 5′-GAGGCATTGCTGATGATCTTGAG-3′. For Western blot, total protein was extracted using RIPA lysis buffer supplemented with protease inhibitors. Protein samples were separated by SDS-PAGE, transferred to PVDF membranes, and probed with anti-CD93 antibody (Wuhan Sanying Biotechnology, Wuhan, China) and anti-GAPDH antibody (loading control). Protein bands were visualized using an enhanced chemiluminescence (ECL) detection kit.

### Quantitative real-time PCR

Total RNA was extracted from samples using Trizol and chloroform, and subsequently reverse-transcribed into cDNA using a reverse transcription kit (TaKaRa, RR036A). Quantitative PCR was performed using PerfectStart Green qPCR SuperMix (TransGen Biotech, AQ601) on Bio-Rad CFX96 Real-Time PCR System with gene-specific primers. The reaction conditions were: 94℃ for 30 seconds, followed by 40 cycles of 94℃ for 5 seconds and 60℃ for 30 seconds. All samples were run in triplicate, and gene expression was normalized to GAPDH and analyzed via the comparative ΔΔCt method.

### Western blot

Protein samples were extracted from cells or tissues using RIPA lysis buffer supplemented with protease inhibitors. The protein concentration was determined using a BCA assay (Beyotime, P0012). Equal amounts of protein (20 µg) were separated by SDS-polyacrylamide gel electrophoresis (SDS-PAGE) and subsequently transferred onto a PVDF membrane. The membrane was blocked with 5% non-fat milk in TBST for 1 hour at room temperature and then incubated overnight at 4℃ with anti-CD93 antibody (Sangon Biotech, D161176) and GAPDH (Proteintech Group, 60004–1-lg). After washing, the membrane was incubated with an appropriate horseradish peroxidase (HRP)-conjugated secondary antibody for 1 hour at room temperature. Protein bands were visualized using an enhanced chemiluminescence (ECL) detection kit (Beyotime, P0018HM), and the signal intensity was quantified by densitometry analysis. Following immunodetection of CD93, the membrane was stripped using a stripping buffer (Beyotime, P0025N), re-blocked, and subsequently probed with an anti-GAPDH antibody. The blot was then incubated with an appropriate secondary antibody and visualized.

### Cell Viability Assay (CCK-8)

Cell viability was assessed using an Enhanced Cell CCK-8 Counting kit (Bioss Biotechnology, BA00208). 1,500 cells per well were seeded in a 96-well plate with three replicate wells per group and allowed to adhere. After cell attachment, the plate was incubated for 4 consecutive days. At each time point (0, 24, 48, and 72 hours), the culture medium was replaced with a mixture of complete medium and CCK-8 reagent (1:9 ratio). To minimize evaporation, 100 µL of PBS was added to the peripheral wells. The plate was then incubated for 30 minutes, and the absorbance at 450 nm was measured using a microplate reader. Wells containing only the CCK-8 medium mixture served as the blank control.

### Cloning formation experiment

Cells from different experimental groups were plated at a low density (1,500 cells per well) in 6-well plates and cultured for 10–14 days to allow colony development, with medium renewal every three days. Upon completion of the culture period, colonies were fixed with 4% paraformaldehyde, washed with PBS, and stained with 0.5% (w/v) crystal violet. After destaining and drying, the number of colonies (defined as clusters of >50 cells) was quantified. The colony formation rate was determined using the formula: Colony Formation Rate (%) = (Number of Colonies/Number of Cells Seeded) × 100%.

### Tube formation assay

A tube formation assay was performed to assess the angiogenic capability of the cells. Pre-chilled Matrigel was carefully pipetted into a 96-well plate (30 µL per well) using cooled pipette tips to prevent premature polymerization. The plate was then incubated at 37℃ for 30 minutes to allow the Matrigel to solidify. Subsequently, 7.5 × 10^4^ cells were seeded onto the polymerized Matrigel in each well and cultured for 6 hours in a 37℃, 5% CO₂ incubator. After incubation, representative images of the tubular structures were captured using an inverted microscope. The formation of capillary-like tubes was quantified by analyzing the images with ImageJ software.

### Detection of permeability of endothelial cells in vitro

In vitro endothelial permeability was evaluated by measuring the flux of FITC-labeled dextran across the cell monolayer. Following a 72-hours co-culture of HUVECs with various gastric adenocarcinoma cell models in Transwell chambers (0.4 µm pores), the inserts were placed into new 24-well plates. The medium in the upper chamber was carefully aspirated and replaced with a solution containing 20 µg/mL FITC-dextran (70 kDa). After a 1-hour incubation, the liquid from the lower chamber was collected. The absorbance of the translocated FITC-dextran was quantified spectrofluorometrically (excitation 490 nm, emission 520 nm) using a microplate reader, and the permeability was calculated accordingly.

### Tumor cell transendothelial migration experiment

Tumor cell transendothelial migration was assessed using a transwell-based assay. After 72 hours of co-culture between HUVECs and different gastric adenocarcinoma cell models in transwell chambers (8 µm pore size), the chambers were transferred to new 24-well plates. Then, 5 × 10^4^ GFP-expressing SGC-7901 cells were seeded into the upper chamber and incubated for 24 hours. Non-migrated cells on the upper surface were gently removed with a cotton swab, and the chambers were rinsed once with pre-warmed culture medium. The migrated GFP-positive cells on the lower side were visualized and imaged under a fluorescence microscope. Quantitative analysis was performed by counting the number of migrated cells using ImageJ software.

### Subcutaneous tumor xenograft in nude mice

All animal studies were conducted using 5-week-old female BALB/c nude mice (Beijing SPF Biotechnology). Mice were randomly divided into experimental and control groups (n = 8 per group). Each mouse received a subcutaneous injection of 5 × 10^6^ SGC-7901 cells (in 100 µL PBS) into the right flank. Tumor growth was monitored every 2–3 days by measuring length (L) and width (W) with a digital caliper; volume was calculated as (L × W^2^)/2. All mice were monitored daily for signs of distress. Predefined humane endpoints were strictly applied, requiring immediate euthanasia (within 1 hour) if mice exhibited: (1) severe lethargy or inability to access food/water; (2) > 20% body weight loss from peak; (3) tumor burden > 1 cm in diameter or ulceration impairing movement; or (4) signs of severe pain (e.g., hunched posture, vocalization). No animals died prior to meeting these criteria. At the experimental endpoint (5 weeks post-inoculation) or upon reaching humane endpoint criteria, mice were euthanized via carbon dioxide asphyxiation in accordance with the American Veterinary Medical Association (AVMA) Guidelines. Briefly, mice were placed in a sealed euthanasia induction chamber, and compressed CO_2_ was introduced at a flow rate of approximately 5.8 L/min (equivalent to displacing 30% of the chamber volume per minute). Flow was maintained until the cessation of respiration, pupillary dilation, and absence of response to mechanical stimuli were confirmed. Gas flow was then stopped, and animals were observed for an additional 2–3 minutes to ensure death. Tumors were then excised, photographed, and weighed. All procedures were performed by researchers trained in laboratory animal care, pain recognition, and humane techniques. The study protocol was approved by the Ethics Committee of Changzhi Medical College (DW2025162) and complied with all relevant guidelines.

### Immunohistochemical (IHC) staining

Tissue samples were fixed in 4% paraformaldehyde, embedded in paraffin, and sectioned into 4−5 µm thick slices. Following deparaffinization and rehydration, antigen retrieval was performed by heating the sections in citrate buffer (pH 6.0). Endogenous peroxidase activity was blocked with 3% hydrogen peroxide. The sections were then incubated overnight at 4℃ with CD31 Polyclonal antibody (Proteintech Group, 11265–1-AP), Alpha smooth muscle actin Polyclonal antibody (Proteintech Group, 14395–1-AP), and Ki-67 Polyclonal antibody (Proteintech Group, 27309–1-AP). After washing, the sections were incubated with an HRP-labeled secondary antibody at room temperature for 1 hour. The antigen-antibody reaction was visualized using a 3, 3’-diaminobenzidine (DAB) substrate, and the nuclei were counterstained with hematoxylin. Finally, the sections were dehydrated, cleared, and mounted. Staining was observed and imaged under a light microscope. Negative controls were processed by omitting the primary antibody.

### Statistical methods

All the above experiments were repeated ≥ 3 times. Data analysis and bar graph generation were performed using Graphpad Prism 9.0 and ImageJ. A *P* value < 0.05 was considered statistically significant. The results of measurement data were expressed as “mean ± standard deviation”, and *t*-*t*est was used for comparison between groups. Chi-square test was used for comparison between groups of count data.

## Results

### CD93 is highly expressed in gastric adenocarcinoma and associated with poor prognosis

**Fig 1:**
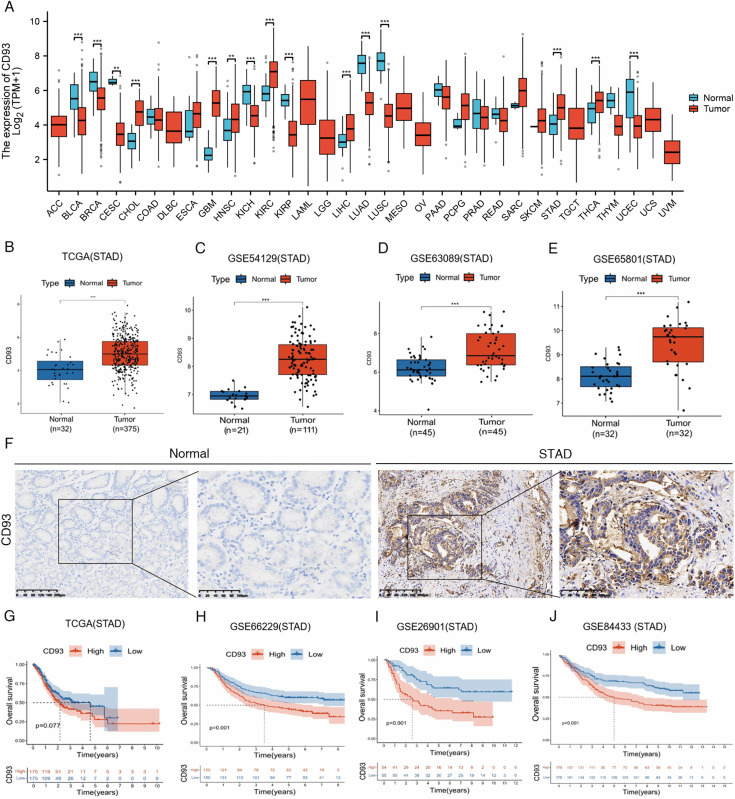
CD93 is highly expressed in gastric adenocarcinoma and associated with poor prognosis. (A) The box plots show the expression levels of CD93 in tumor tissues (red) compared to normal tissues (blue) across multiple common cancer types; (B) The expression levels of CD93 in gastric adenocarcinoma tissues and normal tissues in the TCGA database; (C-E) The expression levels of CD93 in gastric adenocarcinoma tissues and normal tissues in three external independent datasets (GSE54129, GSE63089, GSE65801); (F) Expression of CD93 in normal and tumor tissues was detected by immunohistochemical staining; (G) Survival curve analysis of the TCGA database; (H-J) Survival curve analysis of three external independent datasets (GSE66229, GSE26901, GSE84433). *P < 0.05, **P < 0.01, ***P < 0.001, ****P < 0.0001.

We first conducted a pan-cancer analysis of CD93 expression profiles across multiple tumor types based on the TCGA database, revealing abnormal expression of CD93 in various malignancies. Subsequently, to investigate the role of CD93 in gastric adenocarcinoma, we analyzed CD93 gene expression levels in gastric adenocarcinoma tissues versus normal gastric mucosal epithelial tissues using TCGA database and multiple external datasets (GSE54129 [37], GSE63089 [38], GSE65801 [39]). The results showed that CD93 expression was significantly upregulated in gastric adenocarcinoma tissues compared to normal tissues. Immunohistochemical staining results also supported this finding To further evaluate the relationship between CD93 expression and patient prognosis, we divided gastric adenocarcinoma patients into high-expression and low-expression groups based on CD93 levels and plotted Kaplan-Meier survival curves. The results indicated a trend toward poor prognosis in patients with high CD93 expression within the TCGA cohort, and this association was further confirmed across multiple external GEO datasets, demonstrating that high CD93 expression is significantly linked to adverse outcomes in gastric adenocarcinoma patients(Fig 1A-1J). Collectively, these results suggest that CD93 is significantly upregulated in gastric adenocarcinoma. Moreover, its high expression is positively associated with poor patient prognoses, indicating that CD93 might play a crucial role in the development and progression of gastric adenocarcinoma.

### CD93 promotes proliferation of gastric adenocarcinoma cells

**Fig 2:**
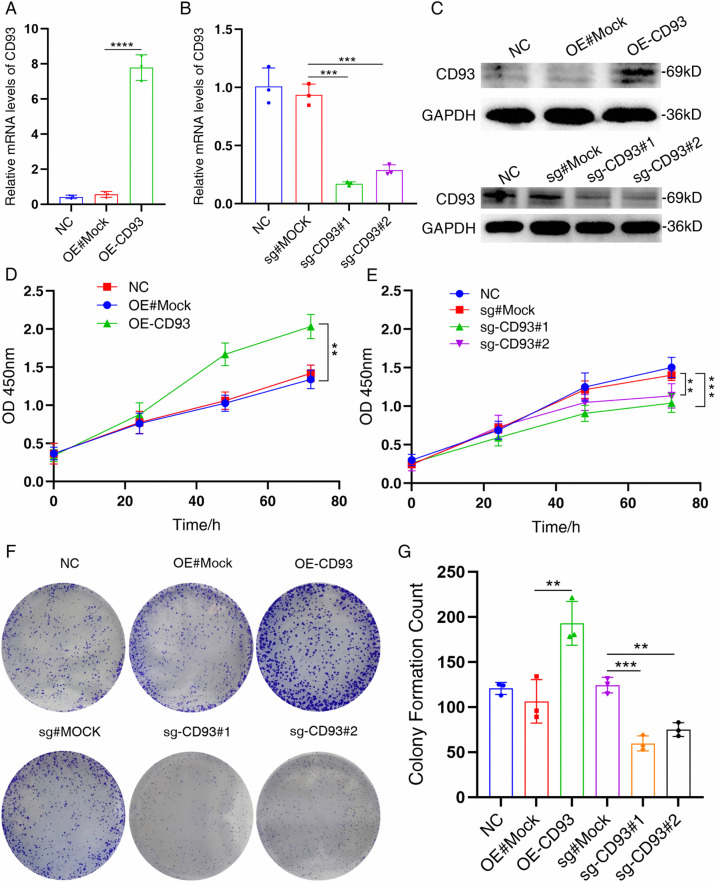
CD93 promotes proliferation of gastric adenocarcinoma cells. (A-C) The expression of CD93 mRNA and protein in the overexpression CD93 and CD93 knockout gastric adenocarcinoma cell models constructed. (D, E) The effects of overexpression and knockout of CD93 on the proliferation of gastric adenocarcinoma cells was detected by CCK-8 assay; (F, G) The effects of overexpression and knockout of CD93 on the proliferation of gastric adenocarcinoma cells and the quantitative graph of the number of tumor cell colonies formed were detected by cloning formation experiment. *P < 0.05, **P < 0.01, ***P < 0.001, ****P < 0.0001.

CD93 is a transmembrane glycoprotein. Previous studies have shown that in nasopharyngeal carcinoma, breast cancer, and osteosarcoma, CD93 can facilitate tumorigenesis and development by enhancing the proliferative capacity of tumor cells [22,31,32]. Nevertheless, whether CD93 can promote the proliferation of gastric adenocarcinoma cells remains unreported in the literature. In order to investigate the impact of CD93 on the malignant biological phenotypes of gastric adenocarcinoma, we first established gastric adenocarcinoma cell models with CD93 overexpression and CD93 knockout. To further investigate the effect of CD93 on the proliferation of gastric adenocarcinoma cells, we conducted CCK-8 proliferation assays to detect the proliferation ability of normal gastric adenocarcinoma cells, CD93 overexpression group and CD93 knockout groups of gastric adenocarcinoma cells respectively. The results indicated that compared with the control group, the proliferation rate of gastric adenocarcinoma cells in the CD93 overexpression group was significantly increased. In contrast, when compared with the control group, the proliferation rate of gastric adenocarcinoma cells in the CD93 knockout groups were significantly decreased, and the difference was statistically significant. Furthermore, the results of the colony formation assay demonstrated that, in comparison to the control group, the number of gastric adenocarcinoma cell colonies formed in the CD93 overexpression group increased significantly, whereas the number of colonies formed in the CD93 knockout group decreased significantly(Fig 2A-2G). This finding was consistent with the trend observed in the CCK-8 assay results.

### CD93 enhances angiogenesis but inhibits transendothelial invasion

To explore the effect of CD93 expression levels in gastric adenocarcinoma cells on angiogenesis, we collected the supernatants of cells from each group following 48 hours of culture. These supernatants were then used to prepare the corresponding conditioned media (CM) for culturing HUVECs in subsequent experiments. The results showed that compared with the control group, the tube formation ability of HUVECs cultured in OE-CD93-CM was significantly increased, while the tube formation ability of HUVECs cultured in sg-CD93#1-CM and sg-CD93#2-CM was significantly decreased, suggesting that high expression of CD93 in gastric adenocarcinoma cells can promote angiogenesis.

**Fig 3:**
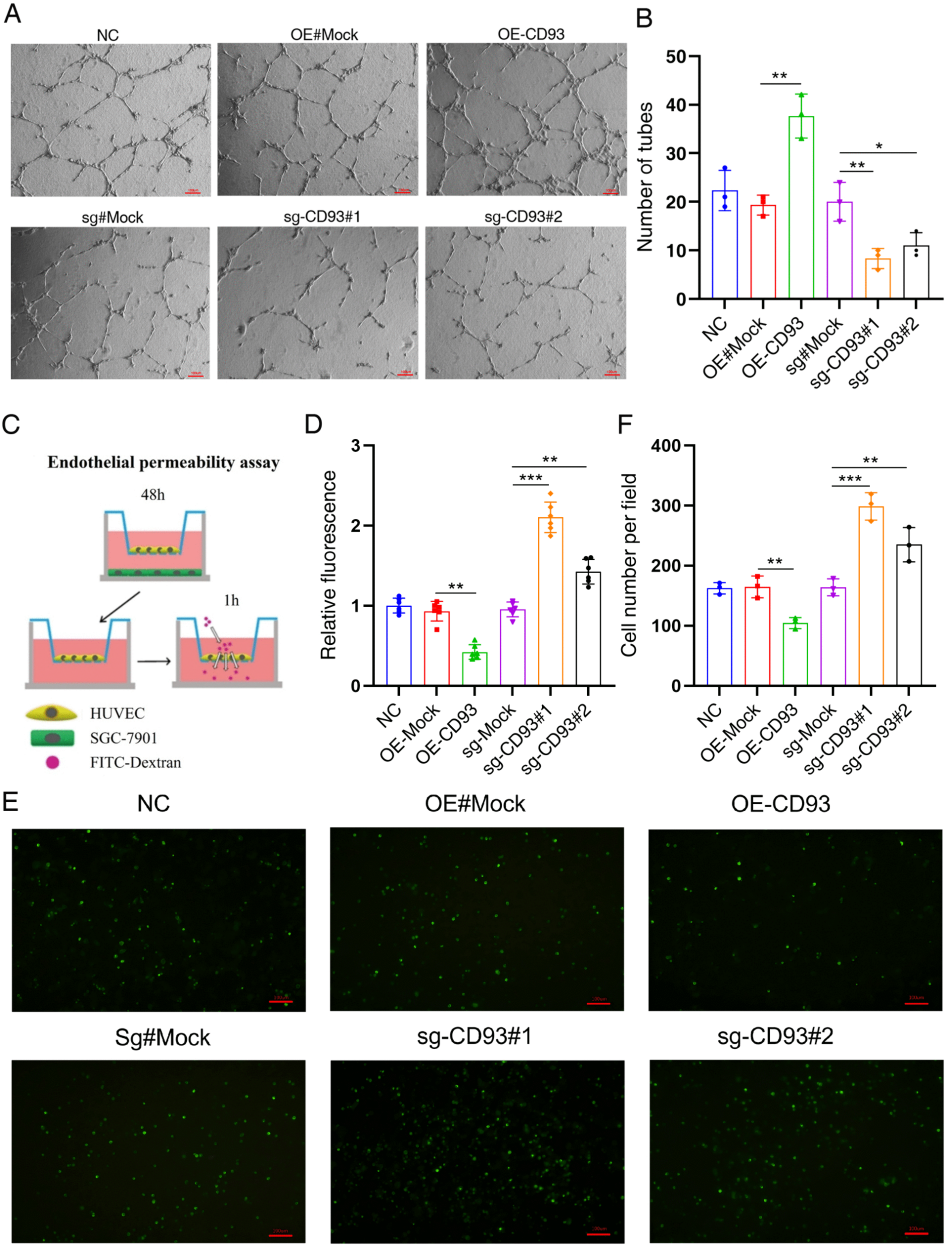
CD93 enhances angiogenesis but inhibits transendothelial invasion. (A, B) Angiogenesis experiments were conducted to detect the effects of CM prepared from overexpressed and knocked-out gastric adenocarcinoma cells on the tube formation ability of HUVECs and to quantify the number of tube-like structures formed by HUVECs. (C) Schematic representation of the SGC-7901/HUVECs co-culture system and endothelial permeability assay. (D) Quantification of monolayer endothelial permeability using FITC-Dextran (70 kDa) tracer. Data represent mean ± SD from three independent experiments. (E) Representative fluorescence micrographs of GFP-labeled gastric adenocarcinoma cells transmigrated through HUVECs monolayers in transwell chambers. (F) Quantitative analysis of invasive GFP-positive gastric adenocarcinoma cells penetrating endothelial monolayers. Statistical significance was determined by one-way ANOVA with Tukey’s post-hoc test (*P < 0.05, **P < 0.01, ***P < 0.001, ****P < 0.0001).

Next, we investigated the effect of CD93 expression in gastric adenocarcinoma cells on the permeability of monolayer vascular endothelial cells. The results showed that gastric adenocarcinoma cells with high expression of CD93 could significantly reduce the permeability of monolayer vascular endothelial cells. Then, we further investigated the effect of high CD93 expression in gastric adenocarcinoma cells on endothelial cells and found that high CD93 expression in gastric adenocarcinoma cells could inhibit the permeability of endothelial monolayers, thereby reducing the ability of gastric adenocarcinoma cells to penetrate vascular endothelium(Fig 3A-3F).

### In vivo effects of CD93 on tumor growth and angiogenesis

To investigate the role of CD93 in gastric tumor growth in vivo, we established a xenograft model by subcutaneously injecting CD93-knockdown or control SGC-7901 cells into the flanks of nude mice. In vivo experiments demonstrated that compared with the control group, the sg-CD93 group exhibited delayed tumor growth, reduced tumor volume, and a significant decrease in tumor weight, suggesting that CD93 knockdown effectively inhibits the growth of xenograft tumors in nude mice.

**Fig 4:**
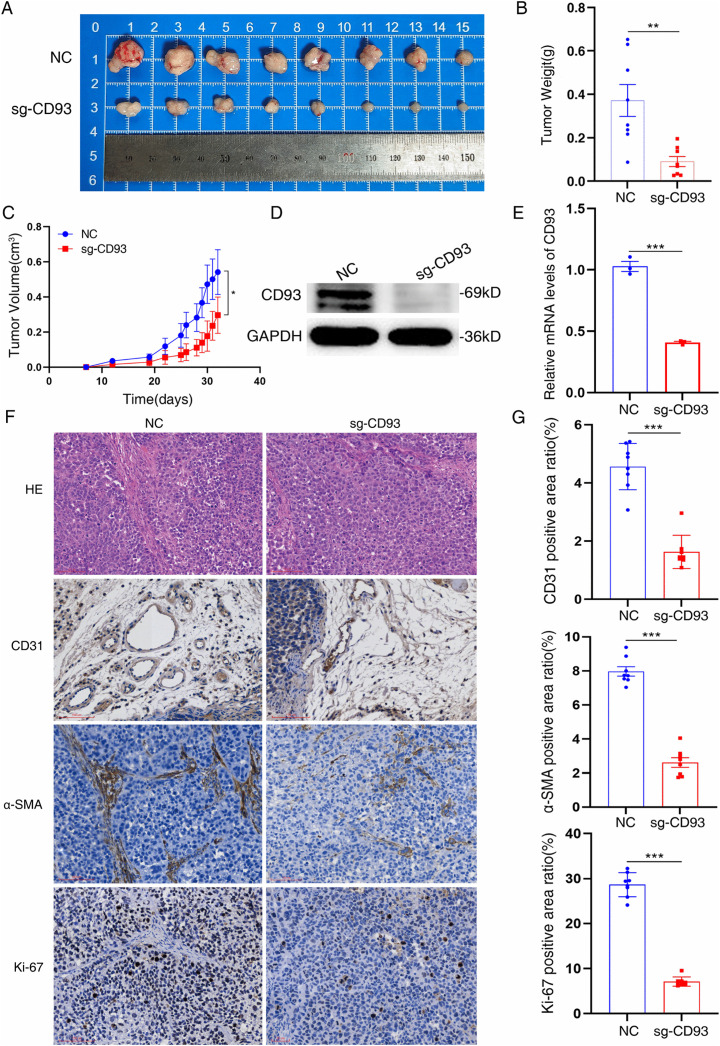
In vivo effects of CD93 on tumor growth and angiogenesis. (A) Representative images of xenograft tumors formed in nude mice subcutaneously injected with SGC-7901 stable cells with CD93 knockdown. (B) Tumor weights measured at the experimental endpoint. (C) Tumor growth curves of each group. (D,E) Knockdown efficiency of CD93 in SGC-7901 cells was verified by WB and qRT-PCR. (F) Expression of the proliferation marker Ki-67, and the endothelial cell marker CD31 in tumor tissues was detected by immunohistochemical staining. (G) Quantitative graphs of Ki-67, CD31 and α-SMA expression in each group of transplanted tumor tissues, with the number of positive cells in three high-power fields (×400) measured respectively. (*P < 0.05, **P < 0.01, ***P < 0.001, ****P < 0.0001).

Furthermore, immunohistochemical staining of the tumor tissues revealed that CD93 knockdown significantly suppressed the expression levels of the proliferation marker Ki-67 and the endothelial marker CD31, indicating impaired tumor cell proliferation and angiogenesis. Moreover, we also found that down-regulation of CD93 could reduce the expression of α-SMA, indicating that CD93 could significantly reduce the permeability of vascular endothelial cells in gastric adenocarcinoma(Fig 4A-4G).

### GO and KEGG enrichment analyses reveal potential roles of CD93 in tumor progression

**Fig 5:**
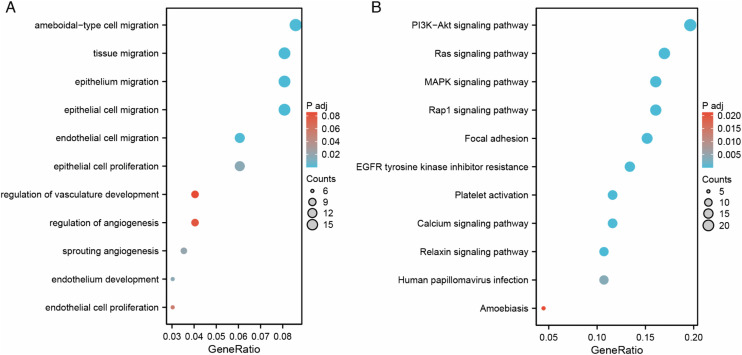
GO and KEGG enrichment analyses reveal potential roles of CD93 in tumor progression. (A) Gene Ontology (GO) enrichment bubble plot of CD93 co‑expressed genes. The x‑axis represents the GeneRatio (number of enriched genes / total background genes in the category). Bubble size indicates the number of enriched genes (Count), and color represents the adjusted p‑value (P.adj), with redder colors denoting higher statistical significance. (B) Kyoto Encyclopedia of Genes and Genomes (KEGG) pathway enrichment bubble plot of CD93 co‑expressed genes. Axes, bubble size, and color are defined as in panel (A).

To investigate the potential molecular mechanisms through which CD93 exerts its biological functions in gastric adenocarcinoma (STAD), we performed Gene Ontology (GO) and Kyoto Encyclopedia of Genes and Genomes (KEGG) enrichment analyses on the set of CD93 co‑expressed genes. As shown in Fig 5, the GO enrichment analysis revealed that CD93 co‑expressed genes were significantly enriched in multiple biological processes related to cell motility and tissue remodeling. Among these, “amoeboidal‑type cell migration” “tissue migration” and “epithelial cell migration” exhibited the highest Gene Ratios. In addition, notable enrichment was observed in angiogenesis‑associated terms, including “regulation of vasculature development” “regulation of angiogenesis” and “endothelial cell proliferation”. These findings suggest that CD93 may be actively involved in tumor cell invasion, metastasis, and angiogenesis.

The subsequent KEGG pathway enrichment analysis identified key signaling networks underlying these observations. CD93 co‑expressed genes were significantly enriched in several classic oncogenic pathways closely related to tumor development and progression, including the PI3K‑Akt signaling pathway, Ras signaling pathway, and MAPK signaling pathway. Furthermore, the analysis also indicated associations between CD93 and platelet activation as well as EGFR tyrosine kinase inhibitor resistance(Fig 5A-5B). Taken together, these enrichment results suggest that CD93 may promote migration, invasion, and angiogenesis in gastric adenocarcinoma cells by activating pro‑oncogenic signaling cascades such as PI3K‑Akt and Ras/MAPK, and may also be involved in drug resistance processes, thereby contributing to disease progression.

## Discussion

Gastric adenocarcinoma is a malignant tumor that originates from the epithelial cells of the gastric mucosa. It is highly invasive and has a strong tendency to metastasize. It has a relatively high incidence and mortality rate in China and Southeast Asia [1,40]. Therefore, clarifying the mechanism of occurrence and development of gastric adenocarcinoma and providing new biomarkers or therapeutic targets for clinical treatment have become urgent problems to be solved. As a transmembrane glycoprotein, CD93 plays a significant role in physiological processes such as cell adhesion, migration and signal transduction, and also plays an important role in the process of vascular development [41]. Currently, multiple studies have shown that CD93 is overexpressed in the tumor vessels of many solid tumors and is closely related to the occurrence and development of various tumors such as osteosarcoma, breast cancer, and nasopharyngeal carcinoma. Patients with high expression of CD93 usually have a poor prognosis [24,35,36,42]. Therefore, we speculate that CD93 may also play the role of an oncogene in the occurrence and development of gastric cancer.

In this study, we first found through the analysis of the TCGA database and multiple external datasets that the expression level of CD93 in gastric adenocarcinoma tissue samples was significantly higher than that in normal gastric mucosal epithelial tissues, and high expression of CD93 was closely related to poor prognosis of patients. To further explore the role of CD93 in the progression of gastric adenocarcinoma, we constructed CD93 knockout and overexpression cell models. The results showed that high expression of CD93 could significantly promote the proliferation of gastric adenocarcinoma cells, suggesting that CD93 may become a potential biomarker for gastric adenocarcinoma.

CD93 is primarily expressed on endothelial cells and is significantly upregulated in tumor-associated blood vessels [43]. However, some studies have shown that CD93 is also co-expressed in various tumor cells, although its exact function and expression levels on the surface of these cells differ [44,45]. CD93 plays a crucial role in tumor angiogenesis by interacting with its ligands—such as polypeptide-2 MMRN2 and insulin-like growth factor binding protein 7 (IGFBP7)—thereby regulating endothelial cell migration, proliferation, and lumen formation [46]. Growing evidence indicates that CD93 plays an important role in angiogenesis and the vascular system across various cancers, including nasopharyngeal carcinoma, glioblastoma, colorectal cancer, and pancreatic ductal adenocarcinoma, and high expression of CD93 correlates with aggressive tumor progression [22,23,29,34]. However, it remains unclear whether changes in CD93 expression levels affect angiogenesis in gastric adenocarcinoma. Our findings demonstrate that upregulated CD93 expression promotes angiogenesis in gastric adenocarcinoma, a phenomenon confirmed in in vivo experiments.

However， the role of CD93 in the regulation of vascular endothelial cell permeability remains controversial. In the study by Vemuri et al., CD93 knockout mice showed increased vascular endothelial cell permeability, suggesting that CD93 may inhibit tumor metastasis by maintaining endothelial barrier function [42].In the study by Sun et al., CD93 knockout mice exhibited decreased vascular endothelial cell permeability [34]. This contradictory result indicates that CD93 may have a complex mechanism and background dependence in the regulation of endothelial cell permeability. To date, no studies have been reported regarding the impact of CD93 on the permeability of vascular endothelial cells in gastric adenocarcinoma. In this study, we found that CD93 reduced the permeability of monolayer endothelial cells and inhibited the invasive ability of tumor cells to penetrate monolayer vascular endothelial cells. Therefore, we speculate that this phenomenon may be related to tumor heterogeneity, but the specific mechanism of action still requires further research.

In addition, anti-angiogenic therapy targeting the VEGF/VEGFR signaling pathway has become a cornerstone in the treatment of multiple solid tumors [47]. However, the clinical benefits of VEGF inhibition are often transient due to intrinsic or acquired resistance, activation of compensatory angiogenic pathways, and persistent abnormalities in tumor vasculature [48]. Increasing evidence suggests that tumor angiogenesis is regulated not only by VEGF-dependent endothelial proliferation, but also by mechanisms involving vascular remodeling, endothelial migration, and microenvironmental interactions [49,50].CD93 has recently emerged as a novel regulator of tumor angiogenesis [33]. Unlike VEGF signaling, which primarily stimulates endothelial cell proliferation and neovascular formation, CD93 appears to be more closely associated with endothelial cell adhesion, migration, and vascular remodeling. As a transmembrane glycoprotein, CD93 plays important roles in physiological processes such as cell adhesion, migration, signal transduction, and vascular development [41]. Previous studies have demonstrated that blockade of CD93 signaling can normalize tumor vasculature and improve vascular integrity, thereby enhancing antitumor immune responses and therapeutic efficacy [34]. In the present study, we observed that CD93 overexpression promoted gastric adenocarcinoma growth and angiogenesis, further supporting its pro-tumorigenic role. Compared with VEGF-targeted therapies, CD93 inhibition may provide additional therapeutic benefits by targeting complementary mechanisms involved in vascular remodeling and tumor microenvironment regulation. Moreover, combined inhibition of CD93 and VEGF pathways may represent a promising strategy to overcome resistance to conventional anti-angiogenic therapies and achieve more durable antitumor effects.

## Conclusion

In conclusion, CD93 is a transmembrane protein closely associated with tumor progression. Our in vitro experiments have confirmed that CD93 promotes the proliferation of gastric adenocarcinoma cells and tumor angiogenesis, thereby contributing to their malignant biological behavior. Importantly, these findings were further validated by our in vivo experiments. However, the specific mechanisms and signaling pathways by which CD93 promotes the progression of gastric adenocarcinoma through influencing angiogenesis remain to be further studied. Our research provides a foundation for understanding the role of CD93 in gastric adenocarcinoma and offers theoretical basis and new ideas for therapeutic targets of gastric adenocarcinoma.

## Supporting information

S1 File Raw images. (PDF)

## Abbreviations

CM: conditioned medium
CCK-8: cell counting kit-8
WB: western blot
FITC-Dextran: fluorescein isothiocyanate-dextran
GFP: green fluorescent protein
HP: helicobacter pylori
HUVECs: human umbilical vein endothelial cells

## References

[1] Bray F, Laversanne M, Sung H, Ferlay J, Siegel RL, Soerjomataram I. Global cancer statistics 2022: GLOBOCAN estimates of incidence and mortality worldwide for 36 cancers in 185 countries. CA: A Cancer J Clin. 2024;74:229–63. doi: 10.3322/caac.21834 38572751

[2] Smyth EC, Nilsson M, Grabsch HI, van Grieken NC, Lordick F. Gastric cancer. Lancet. 2020;396(10251):635–48. doi: 10.1016/S0140-6736(20)31288-5 32861308

[3] Yan L, Chen Y, Chen F, Tao T, Hu Z, Wang J, et al. Effect of Helicobacter pylori eradication on gastric cancer prevention: updated report from a randomized controlled trial with 26.5 years of follow-up. Gastroenterology. 2022;163(1):154-162.e3. doi: 10.1053/j.gastro.2022.03.039 35364066

[4] W X, T Y, J Z, Z M, W Y, Z H, et al. Chinese and global burdens of gastrointestinal cancers from 1990 to 2019. Frontiers in Public Health. 2022;10. doi: 10.3389/fpubh.2022.941284 PMC9326121 35910886

[5] Wu S, Xu P, Zhang F. Advances in targeted therapy for gastric cancer based on tumor driver genes. Zhejiang Da Xue Xue Bao Yi Xue Ban. 2023;53(1):73–83. doi: 10.3724/zdxbyxb-2023-0522 38413217 PMC10938109

[6] Yan HHN, Siu HC, Law S, Ho SL, Yue SSK, Tsui WY, et al. A comprehensive human gastric cancer organoid biobank captures tumor subtype heterogeneity and enables therapeutic screening. Cell Stem Cell. 2018;23(6):882-897.e11. doi: 10.1016/j.stem.2018.09.016 30344100

[7] Ajani JA, D’Amico TA, Bentrem DJ, Chao J, Cooke D, Corvera C. Gastric cancer, version 2.2022, NCCN clinical practice guidelines in oncology. J Natl Compr Cancer Netw. 2022;20:167–92. doi: 10.6004/jnccn.2022.0008 35130500

[8] Hinshaw DC, Shevde LA. The tumor microenvironment innately modulates cancer progression. Cancer Res. 2019;79(18):4557–66. doi: 10.1158/0008-5472.CAN-18-3962 31350295 PMC6744958

[9] Xiao Y, Yu D. Tumor microenvironment as a therapeutic target in cancer. Pharmacol Ther. 2021;221:107753. doi: 10.1016/j.pharmthera.2020.107753 33259885 PMC8084948

[10] Lamplugh Z, Fan Y. Vascular microenvironment, tumor immunity and immunotherapy. Front Immunol. 2021;12:811485. doi: 10.3389/fimmu.2021.811485 34987525 PMC8720970

[11] Liu Z, Liu L, Guo C, Yu S, Meng L, Zhou X, et al. Tumor suppressor gene mutations correlate with prognosis and immunotherapy benefit in hepatocellular carcinoma. Int Immunopharmacol. 2021;101(Pt B):108340. doi: 10.1016/j.intimp.2021.108340 34789428

[12] Brown TP, Ganapathy V. Lactate/GPR81 signaling and proton motive force in cancer: role in angiogenesis, immune escape, nutrition, and Warburg phenomenon. Pharmacol Ther. 2020;206:107451. doi: 10.1016/j.pharmthera.2019.107451 31836453

[13] Wang L, Zhang W, Zhang J, Zheng M, Pan X, Guo H, et al. Inhibitory effect of adenosine on adaptive antitumor immunity and intervention strategies. Zhejiang Da Xue Xue Bao Yi Xue Ban. 2023;52(5):567–77. doi: 10.3724/zdxbyxb-2023-0263 37916308 PMC10630057

[14] He Q, Ye A, Ye W, Liao X, Qin G, Xu Y, et al. Cancer-secreted exosomal miR-21-5p induces angiogenesis and vascular permeability by targeting KRIT1. Cell Death Dis. 2021;12(6):576. doi: 10.1038/s41419-021-03803-8 34088891 PMC8178321

[15] Hu JL, Wang W, Lan XL, Zeng ZC, Liang YS, Yan YR, et al. CAFs secreted exosomes promote metastasis and chemotherapy resistance by enhancing cell stemness and epithelial-mesenchymal transition in colorectal cancer. Mol Cancer. 2019;18(1):91. doi: 10.1186/s12943-019-1019-x 31064356 PMC6503554

[16] Ili C, Buchegger K, Demond H, Castillo-Fernandez J, Kelsey G, Zanella L, et al. Landscape of genome-wide DNA methylation of colorectal cancer metastasis. Cancers (Basel). 2020;12(9):2710. doi: 10.3390/cancers12092710 32971738 PMC7564781

[17] Kao Y-C, Jiang S-J, Pan W-A, Wang K-C, Chen P-K, Wei H-J, et al. The epidermal growth factor-like domain of CD93 is a potent angiogenic factor. PLoS One. 2012;7(12):e51647. doi: 10.1371/journal.pone.0051647 23272129 PMC3525571

[18] Zhang C, Nan X, Zhang B, Wu H, Zeng X, Song Z, et al. Blockade of CD93 in pleural mesothelial cells fuels anti-lung tumor immune responses. Theranostics. 2024;14(3):1010–28. doi: 10.7150/thno.89144 38250037 PMC10797298

[19] Qu J, Lin L, Fu G, Zheng M, Geng J, Sun X, et al. The analysis of multiple omics and examination of pathological images revealed the prognostic and therapeutic significances of CD93 in lung squamous cell carcinoma. Life Sci. 2024;339:122422. doi: 10.1016/j.lfs.2024.122422 38224815

[20] Shen Y, Wu Y, Hao M, Fu M, Zhu K, Luo P, et al. Clinicopathological association of CD93 expression in gastric adenocarcinoma. J Cancer Res Clin Oncol. 2024;150(8):400. doi: 10.1007/s00432-024-05874-4 39190192 PMC11349802

[21] Wu B, Fu L, Guo X, Hu H, Li Y, Shi Y, et al. Multi-omics profiling and digital image analysis reveal the potential prognostic and immunotherapeutic properties of CD93 in stomach adenocarcinoma. Front Immunol. 2023;14:984816. doi: 10.3389/fimmu.2023.984816 36761750 PMC9905807

[22] Bao L, Tang M, Zhang Q, You B, Shan Y, Shi S, et al. Elevated expression of CD93 promotes angiogenesis and tumor growth in nasopharyngeal carcinoma. Biochem Biophys Res Commun. 2016;476(4):467–74. doi: 10.1016/j.bbrc.2016.05.146 27255994

[23] Olsen RS, Lindh M, Vorkapic E, Andersson RE, Zar N, Löfgren S, et al. CD93 gene polymorphism is associated with disseminated colorectal cancer. Int J Colorectal Dis. 2015;30(7):883–90. doi: 10.1007/s00384-015-2247-1 26008729 PMC4471320

[24] Zhu W, Yi Q, Wang J, Ouyang X, Yang K, Jiang B, et al. Comprehensive analysis of CLEC family genes in gastric cancer prognosis immune response and treatment. Sci Rep. 2025;15(1):5956. doi: 10.1038/s41598-024-80204-9 39966377 PMC11836380

[25] Riether C, Radpour R, Kallen NM, Bürgin DT, Bachmann C, Schürch CM, et al. Metoclopramide treatment blocks CD93-signaling-mediated self-renewal of chronic myeloid leukemia stem cells. Cell Rep. 2021;34(4):108663. doi: 10.1016/j.celrep.2020.108663 33503440

[26] Kinstrie R, Horne GA, Morrison H, Irvine D, Munje C, Castañeda EG, et al. CD93 is expressed on chronic myeloid leukemia stem cells and identifies a quiescent population which persists after tyrosine kinase inhibitor therapy. Leukemia. 2020;34(6):1613–25. doi: 10.1038/s41375-019-0684-5 31896780 PMC7272220

[27] Jia J, Liu B, Wang D, Wang X, Song L, Ren Y, et al. CD93 promotes acute myeloid leukemia development and is a potential therapeutic target. Exp Cell Res. 2022;420(2):113361. doi: 10.1016/j.yexcr.2022.113361 36152731

[28] Han H, Liu J, Zhu S, Zhao T. Identification of two key biomarkers CD93 and FGL2 associated with survival of acute myeloid leukaemia by weighted gene co-expression network analysis. J Cell Mol Med. 2024;28(14):e18552. doi: 10.1111/jcmm.18552 39054581 PMC11272607

[29] Langenkamp E, Zhang L, Lugano R, Huang H, Elhassan TEA, Georganaki M, et al. Elevated expression of the C-type lectin CD93 in the glioblastoma vasculature regulates cytoskeletal rearrangements that enhance vessel function and reduce host survival. Cancer Res. 2015;75(21):4504–16. doi: 10.1158/0008-5472.CAN-14-3636 26363010

[30] Ma K, Chen S, Chen X, Zhao X, Yang J. CD93 is associated with glioma-related malignant processes and immunosuppressive cell infiltration as an inspiring biomarker of survivance. J Mol Neurosci. 2022;72(10):2106–24. doi: 10.1007/s12031-022-02060-4 36006582 PMC9596571

[31] Zhang Y, Liu Y, Ma Y, Xu Y, Wang G, Han X. CD93 aggravates cell proliferation, angiogenesis and immune escape in osteosarcoma through triggering the PI3K/AKT pathway. J Orthop Sci. 2025;30(4):685–91. doi: 10.1016/j.jos.2024.10.011 39542800

[32] Liu H, Zhang J, Zhao Y, Fan Z, Yang Y, Mao Y, et al. CD93 regulates breast cancer growth and vasculogenic mimicry through the PI3K/AKT/SP2 signaling pathway activated by integrin β1. J Biochem Mol Toxicol. 2024;38(4):e23688. doi: 10.1002/jbt.23688 38511888

[33] Masiero M, Simões FC, Han HD, Snell C, Peterkin T, Bridges E, et al. A core human primary tumor angiogenesis signature identifies the endothelial orphan receptor ELTD1 as a key regulator of angiogenesis. Cancer Cell. 2013;24(2):229–41. doi: 10.1016/j.ccr.2013.06.004 23871637 PMC3743050

[34] Sun Y, Chen W, Torphy RJ, Yao S, Zhu G, Lin R, et al. Blockade of the CD93 pathway normalizes tumor vasculature to facilitate drug delivery and immunotherapy. Sci Transl Med. 2021;13(604):eabc8922. doi: 10.1126/scitranslmed.abc8922 34321321 PMC8749958

[35] Jiang Q, Kuai J, Jiang Z, Que W, Wang P, Huang W, et al. CD93 overexpresses in liver hepatocellular carcinoma and represents a potential immunotherapy target. Front Immunol. 2023;14:1158360. doi: 10.3389/fimmu.2023.1158360 37483608 PMC10359974

[36] Tosi GM, Caldi E, Parolini B, Toti P, Neri G, Nardi F, et al. CD93 as a potential target in neovascular age-related macular degeneration. J Cell Physiol. 2017;232(7):1767–73. doi: 10.1002/jcp.25689 27859225

[37] Liu B, Zhu Z, Yan M, Li J, Zhang J, Li C. Global gene expression analysis of gastric cancer by oligonucleotide microarrays. 2017. https://www.ncbi.nlm.nih.gov/geo/query/acc.cgi?acc=GSE54129

[38] Zhang X, Ni Z, Duan Z, Xin Z, Wang H, Tan J, et al. Overexpression of E2F mRNAs associated with gastric cancer progression identified by the transcription factor and miRNA co-regulatory network analysis. PLoS One. 2015;10(2):e0116979. doi: 10.1371/journal.pone.0116979 25646628 PMC4315469

[39] Li H, Yu B, Li J, Su L, Yan M, Zhang J, et al. Characterization of differentially expressed genes involved in pathways associated with gastric cancer. PLoS One. 2015;10(4):e0125013. doi: 10.1371/journal.pone.0125013 25928635 PMC4415781

[40] Yang H, Zhou S, Wang W, Zhao Y, Qiu Y, Jiang X, et al. The trends of gastric cancer in china from 1990 to 2019 and predictions to 2040: a bayesian age-period-cohort prediction study. Cancer Control. 2024;31:10732748241293982. doi: 10.1177/10732748241293982 39420585 PMC11489932

[41] Khan KA, McMurray JL, Mohammed F, Bicknell R. C-type lectin domain group 14 proteins in vascular biology, cancer and inflammation. FEBS J. 2019;286(17):3299–332. doi: 10.1111/febs.14985 31287944 PMC6852297

[42] Vemuri K, de Alves Pereira B, Fuenzalida P, Subashi Y, Barbera S, van Hooren L, et al. CD93 maintains endothelial barrier function and limits metastatic dissemination. JCI Insight. 2024;9(7):e169830. doi: 10.1172/jci.insight.169830 38441970 PMC11128212

[43] Tossetta G, Piani F, Borghi C, Marzioni D. Role of CD93 in health and disease. Cells. 2023;12(13):1778. doi: 10.3390/cells12131778 37443812 PMC10340406

[44] Zhang Z, Zheng M, Ding Q, Liu M. CD93 correlates with immune infiltration and impacts patient immunotherapy efficacy: a pan-cancer analysis. Front Cell Dev Biol. 2022;10:817965. doi: 10.3389/fcell.2022.817965 35242761 PMC8886047

[45] Guo A, Zhang J, Tian Y, Peng Y, Luo P, Zhang J, et al. Identify the immune characteristics and immunotherapy value of CD93 in the pan-cancer based on the public data sets. Front Immunol. 2022;13:907182. doi: 10.3389/fimmu.2022.907182 36389798 PMC9646793

[46] Barbera S, Lugano R, Pedalina A, Mongiat M, Santucci A, Tosi GM, et al. The C-type lectin CD93 controls endothelial cell migration via activation of the Rho family of small GTPases. Matrix Biol. 2021;99:1–17. doi: 10.1016/j.matbio.2021.05.006 34062268

[47] Ellis LM, Hicklin DJ. VEGF-targeted therapy: mechanisms of anti-tumour activity. Nat Rev Cancer. 2008;8(8):579–91. doi: 10.1038/nrc2403 18596824

[48] Bergers G, Hanahan D. Modes of resistance to anti-angiogenic therapy. Nat Rev Cancer. 2008;8(8):592–603. doi: 10.1038/nrc2442 18650835 PMC2874834

[49] De Palma M, Biziato D, Petrova TV. Microenvironmental regulation of tumour angiogenesis. Nat Rev Cancer. 2017;17(8):457–74. doi: 10.1038/nrc.2017.51 28706266

[50] Carmeliet P, Jain RK. Principles and mechanisms of vessel normalization for cancer and other angiogenic diseases. Nat Rev Drug Discov. 2011;10(6):417–27. doi: 10.1038/nrd3455 21629292

