# Supplementary material for: Effects of high CD93 expression on tumor growth and angiogenesis in gastric adenocarcinoma
Source: PLoS One. 2026 Aug 12;21(8):e0355284. doi: 10.1371/journal.pone.0355284 (PMC13465816; doi:10.1371/journal.pone.0355284)
Supplement: Supplementary file 1 [file pone.0355284.s001.pdf]

Figure 2 CD93 promotes proliferation of gastric adenocarcinoma cells.

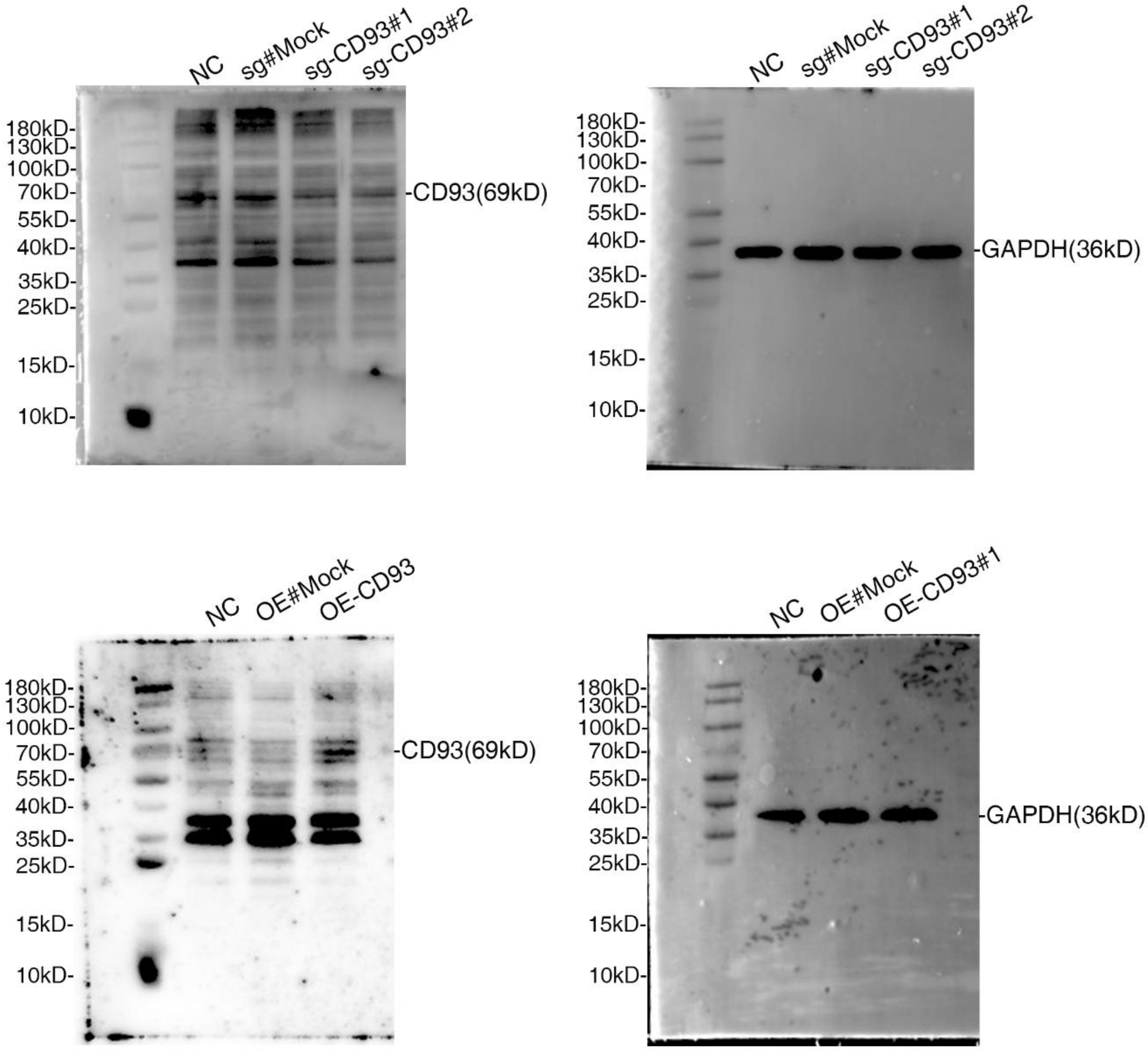

Figure 4 In vivo effects of CD93 on tumor growth and angiogenesis.

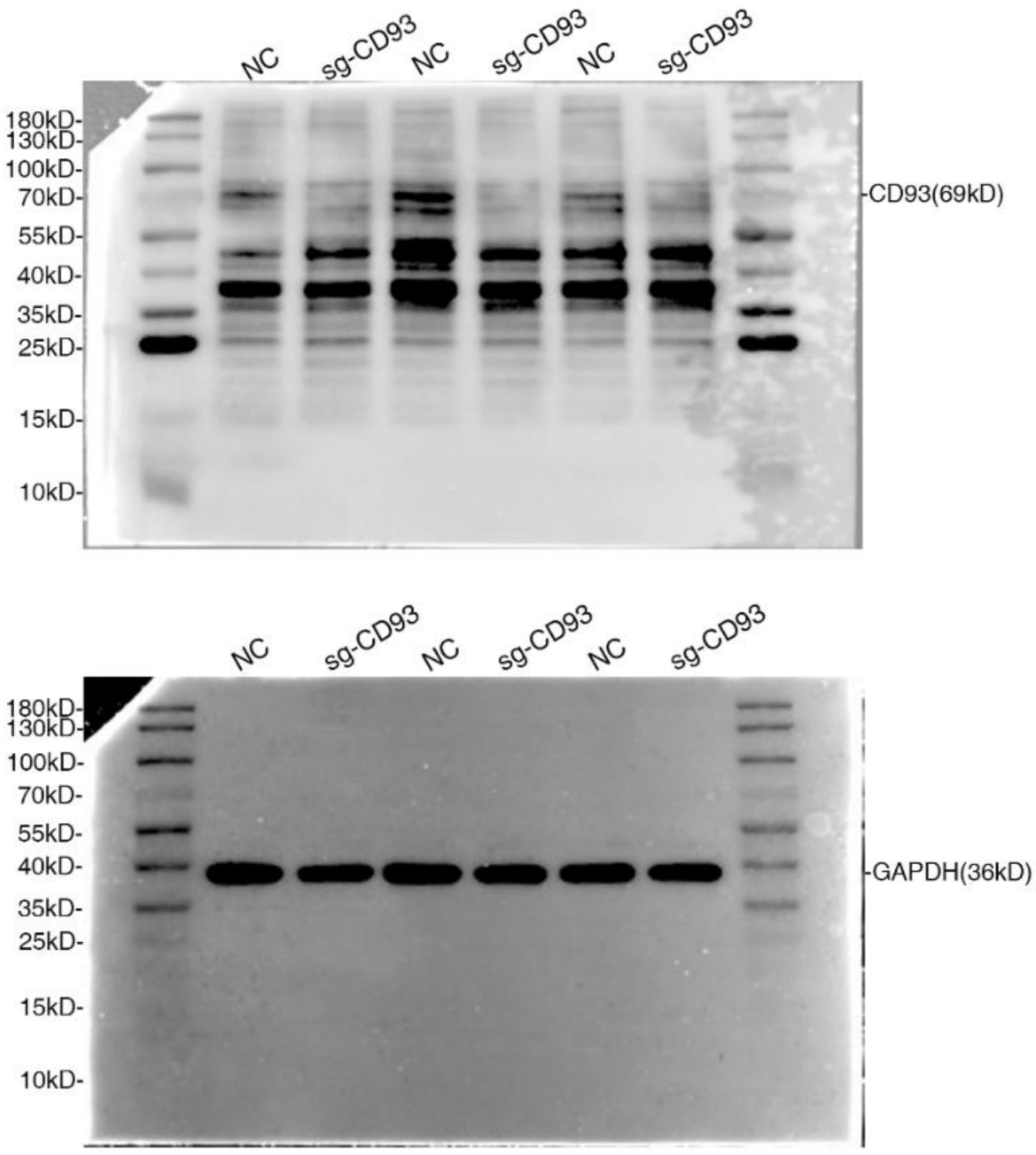

Protein samples were extracted from cells or tissues using RIPA lysis buffer supplemented with protease inhibitors. The protein concentration was determined using a BCA assay (Beyotime, P0012). Equal amounts of protein (20μg) were separated by SDS-polyacrylamide gel electrophoresis (SDS-PAGE) and subsequently transferred onto a PVDF membrane. The membrane was blocked with 5% non-fat milk in TBST for 1 hour at room temperature and then incubated overnight at 4°C with anti-CD93 antibody (Sangon Biotech, D161176) and GAPDH (Proteintech Group, 60004-1-lg). After washing, the membrane was incubated with an appropriate horseradish peroxidase (HRP)-conjugated secondary antibody for 1 hour at room temperature. Protein bands were visualized using an enhanced chemiluminescence (ECL) detection kit (Beyotime, P0018HM), and the signal intensity was quantified by densitometry analysis.Following immunodetection of CD93, the membrane was stripped using a stripping buffer (Beyotime, P0025N), re-blocked, and subsequently probed with an anti-GAPDH antibody. The blot was then incubated with an appropriate secondary antibody and visualized.
